# Coke Poisoning in a Church

**Published:** 1856-04

**Authors:** John Davy


### ORIGINAL COMMUNICATIONS.

### COKE POISONING IN A CHURCH:

### NOTICE OF THE NOXIOUS EFFECTS OF EFFLUVIA FROM A COKE-FIRE ESCAPING INTO A CHURCH DURING SERVICE.

By JOHN DAVY, M.D., F.R.S.

THE noxious effects I am about to describe, with the hope of supplying a preventive lesson, occurred in the new church in Ambleside, on Sunday, January 6th, during the morning service, and were unquestionably due to the escape of mixed gases, evolved from ignited coke used in the air-heating apparatus designed for warming the building.

As preliminary, I may mention that the church is of such a size as to have sittings without galleries for above nine hundred persons; that it is lofty, open to the very rafters, and of a capacity equal to about 150,000 cubic feet. It may be further right to state that the warming apparatus consists of a stove, placed in a crypt under the chancel at the east end, and of a single flue, communicating with the open air, and running through the basement floor of the building under the middle aisle, in which are three grated openings for allowing the air, heated by a cockle, to pass; two of which, the more western ones, were open; the third, the eastern, closed. In consequence of the apparatus affording an inadequate supply of heated air, precautions were taken to confine it, and exclude the cold air; the windows were all closed, as were also the doors, after the commencement of the service, only one having been opened previously, and that to leeward.

At the time, the atmosphere was in a state not favourable to the diffusion and dispersion of smoke or vapour, but rather to its stagnation and accumulation: the sky was overcast with dark, low clouds; the little wind that there was, was southerly; and so mild was the day, that bats and insects were abroad, and were seen on the wing between one and two o'clock in the afternoon.

I proceed now to the effects. At the commencement of the service there was an unpleasant smell perceived, like that from coal-tar, or of smoke from an ill burning fire; and when the sun shone, which it did at short intervals, its light was peculiar, from the hazy quality of the vaporous air. But no apprehension was felt of anything injurious, nor was any alarm excited, till towards the end of the communion service; when, one after another, children and young people began to go out from feeling unwell, the numbers rapidly increasing, till, shortly after the commencement of the sermon, the alarm became so general, almost amounting to a panic, that the minister thought it necessary to abruptly bring his discourse to a termination; when, though there was no rush to the open air, there was no delay on the part of any one present from seeking it. Of the scene outside, from the many sufferers, some prostrate, some in danger of life, and variously affected by the noxious air they had breathed, it would be difficult to give an idea. Hardly a person, out of a congregation probably of four hundred at least, did not feel more or less unwell, or was not alarmed on account of a child or near relation seriously affected. Nor was the alarm confined to the spot and the witnesses and sufferers there; it presently spread to the town,—one of the first who spoke of what had happened saying that "all the people in the church were dead":—a good example, I may remark, of the exaggeration that is sure to be run into on an occasion of the kind. And it was not the only one: even the accounts which appeared in the papers, given by persons writing from Ambleside, were not free from gross mistakes, showing how little trust is to be placed in what is hastily published of passing events.

I shall attempt now, from such information as I have been able to collect, especially from Mr. Wm. Fell, the esteemed surgeon of the place, who zealously gave his assistance on the occasion, to describe the principal symptoms of those who suffered. I have already mentioned that scarcely any one altogether escaped suffering. The most robust, of mature age, of both sexes, experienced least bad effect; little more than headache of some hours duration. Those who experienced the worst effects, were children, and young delicate women. Vomiting was a common symptom in the former, and was attended with great prostration of strength and feebleness of the heart's action, and a tendency to fainting. Those who threw up the contents of their stomachs were the soonest to recover. Tremors of the hands and feet, with diminished sensation, threatening paralysis, occurred in many instances of the latter. Oppressed breathing, with uneasiness or pain of chest, was pretty commonly experienced. Next to the very young and delicate, those advanced in years and the plethoric seemed to be most affected.

Apart from age and constitution, position—that is, in relation to the openings of the flue—was not a matter of indifference in relation to the severity of the symptoms; those suffering most who were nearest the openings, especially at the west end, where the majority of the children, those belonging to the Sunday school, were seated, not in pews, but on open forms, so that nothing screened them from the flow of the vapour in their direction. That there was an accumulation of the noxious agents in this portion of the church, was indicated in a visible manner when the doors which are towards the west end were thrown open, by the stream of thick misty air which then rushed out.

Of those affected, the greater number were pretty well before the following day. In a very few instances, the indisposition produced continued—but gradually diminishing—for several days. In one of the most severe cases, a young lady of about seventeen, the recovery was not complete for nearly a week, and hardly then. She fainted in the attempt to walk home, was afterwards hysterically convulsed, had a feeling of extreme feebleness and languor, with oppression and pain of chest, and loss of appetite. In a large number of other instances, something similar occurred. The maximum of noxious effects was experienced after leaving the church and going into the open air.

Of the organs affected, the lungs, the heart, and the nervous system, appeared to bear the brunt of the effect. In no case that I could hear of were the bowels deranged; and the stomach probably was only sympathetically so, and so also the voluntary muscles. In the only instance that I heard described, in which attention was given to the premonitory symptoms, the progress of the morbid action, it was that of a delicate boy aged about twelve; his mother, who sat by him, noticed his incessant yawning, and this for a considerable time before he was taken ill, when he became so ill and suddenly enfeebled that he required to be carried out.

I have said that the effects were unquestionably owing to the escape of the mixed gases produced by the ignited coke, the fuel used in the stove. The evidence on this point is very clear. There was no other source of the noxious effluvia; and that the coke employed was of a quality capable of yielding noxious gases in burning, other than carbonic acid, might even *à priori* be inferred, inasmuch as it burnt with flame; a fact of which I had assurance from the man who attended the stove, and which I have confirmed by trial, using small portions of the identical fuel; these I found, especially the heavier kind, to burn when ignited with a pale blue lambent flame, similar to that of carbonic oxide.*

Some other gases might have been produced at the same time, such as carburetted hydrogen, and sulphurous acid gas; but it is not necessary to insist on these, as their presence can only be conjectured. If present, they could only be in very minute quantity, especially the latter; for I did not perceive the odour of it, nor was cough one of the common symptoms of those seriously affected.

Moreover, that carbonic oxide and carbonic acid were the gases chiefly concerned, seems to be pretty certain from the symptoms, were these considered alone. I shall give some extracts from the writings of those who have made trial of the more deleterious one, carbonic oxide, in order to impress the danger more strongly on those ignorant of the science of the subject. Sir H. Davy, in his *Elements of Chemical Philosophy*, states that "carbonic oxide may be taken into the lungs, but is fatal to animal life": adding, "I once took three inspirations of it, mixed with about one-fourth of common air; the effect was a temporary loss of sensation, which was succeeded by giddiness, sickness, acute pains in different parts of the body, and extreme debility: some days elapsed before I entirely recovered." According to Clement and Desormes, it produces when inspired giddiness and fainting fits.† They found that birds put into this gas "dropped down dead before they had time to take them out."‡ When breathed nearly pure, its intensely noxious effects are well displayed in the following quotation, in which are described most of the symptoms experienced by the several individuals who suffered on the occasion under consideration. Sir H. Davy says: "I made three inspirations and expirations of the hydrocarbonate (so carbonic oxide was then called). The first inspiration produced a sort of numbness and loss of feeling in the chest and about the pectoral muscles. After the second inspiration, I lost all power of perceiving external things, and had no distinct sensation except a terrible oppression on the chest. During the third inspiration, this feeling disappeared; I seemed sinking into annihilation, and had

---

* Probably this gas is always produced when coke is used in fires with excess of fuel, and will escape unburnt if the surface fire be not powerful. Carbon, it is well known, acting on carbonic acid at a high temperature, takes from it one proportion of its oxygen, converting it into carbonic oxide, so that two volumes of the latter take the place of one of the former.

† GMELIN'S Handbook of Chemistry, vol. ii, p. 89.

‡ THOMSON'S System of Chemistry, 5th ed., vol. ii, p. 24.

just power enough to drop the mouth-piece from my unclosed lips. A short interval must have passed, during which I respired common air, before the objects about me were distinguishable. On recollecting myself, I faintly articulated, ‘ I do not think I shall die’. Putting my finger on the wrist, I found my pulse thread-like, and beating with excessive quickness. In less than a minute I was able to walk, and the painful oppression on the chest directed me to the open air. After making a few steps, which carried me to the garden, my head became giddy, my knees trembled, and I had just sufficient voluntary power to throw myself on the grass. Here the painful feeling of the chest increased with such violence as to threaten suffocation. At this moment, I asked for some nitrous oxide. Mr. Dwyer brought me a mixture of oxygen and nitrous oxide. I breathed this for a minute, and *believed* myself relieved. In five minutes, the painful feelings began gradually to diminish. In an hour they had nearly disappeared, and I felt only excessive weakness, and a slight swimming of the head. My voice was very feeble and indistinct. I afterwards walked slowly for about half an hour with Mr. Tobin, jun.; and, on my return, was so much stronger and better, as to believe that the effects of the gas had disappeared, though my pulse was 120, and very feeble. I continued without pain for near three-quarters of an hour, when the giddiness returned with such violence as to oblige me to lie on the bed; it was accompanied with nausea, loss of memory, and deficient sensation. In about an hour and a half the giddiness went off, and was succeeded by an excruciating pain in the forehead and between the eyes, with transient pains in the chest and extremities. Towards night, these affections gradually diminished; at ten, no disagreeable feeling except weakness remained.”*

Of the ascertained effects of carbonic acid I need not be so particular, as they are so well known. Who has not heard of the too often repeated experiments in the Grotto del Cane? I may briefly mention the trials which Sir H. Davy made of it on himself. He attempted to breathe the gas pure, and also diluted with common air. Unless considerably diluted, he found that it occasioned a convulsive closure of the glottis, preventing its admission into the lungs. He breathed, he informs us, a mixture of three quarts of carbonic acid and seven of common air, for near a minute. “At the time, it produced a slight degree of giddiness, and an inclination to

* Researches in Collected Works, vol. iii, p. 279.

sleep; effects," he adds, "which rapidly disappeared after he had ceased to respire it, without being followed by any other affections."

As to the manner in which the noxious gases got admittance into the air-flue, a conjecture only can be offered, as no examination has yet been made of the apparatus, and cannot be made without laying open the stove. The great probability is that fissures have formed in the brickwork, and that through them the gases passed from the fire into the air-flue; indeed, there seems no other mode of explaining the accident.

In giving the above account, I have been minute in consideration of the importance of the subject—that of warming public buildings, and the too little attention commonly paid by architects to a matter, as we have seen, involving risk of life. In the contract for building the Ambleside church, the mode of warming it was not even noticed in the specifications; and though the flues were made under the superintendence of the clerk of the works, it was without the knowledge of his principal. Further, in illustration of the want of due attention to this important object, I may mention that, owing to the chimney of the stove and of the vestry fire-place terminating above the belfry and in the open vault of the spire, the ringers on more than one occasion have been sufferers from the noxious gases descending on them, which could hardly fail of happening in a calm state of the atmosphere.

In alluding to the want of precaution as regards obtaining artificial warmth by fires, the remark is of very wide application; and owing, undoubtedly, to ignorance as much as to carelessness. Patent fuel is advertised, fit, as vaunted, for use in stoves, in passages and rooms without chimneys, as if the carbonic acid gas produced were respirable and innocent; and it is no doubt bought and employed with that belief. How often do we hear of lives sacrificed from burning charcoal in huts and tents! It was one of the many causes in operation last winter in the destruction of life amongst our troops before Sebastopol, which ordinary care and science should have excluded.

It may perhaps be said, that such fatal accidents are few in comparison with the use made of charcoal fires. That is true; because, where such fires are most used, as in the East, the wooden houses are nowise air-tight, and consequently allow a degree of ventilation, preventing accumulation of the noxious airs. Moreover, in the East, the charcoal fire is never brought into a room till the coal has been well ignited, and it has ceased, as it may be inferred, to produce carbonic oxide.

In conclusion, let me remark, if such morbid effects as I have described, endangering life, can take place in a church so capacious as that of Ambleside, and holding at the time less than one-half its full complement of people, how much greater must be the risk, when a like mode of heating is employed in smaller, more confined, and crowded buildings, whether public or private.

Lesketh How, Ambleside, Jan. 12, 1856.

1. The DISEASES* of colliery operatives:

